# Non-obstructive azoospermia: current and future perspectives

**DOI:** 10.12703/r/10-7

**Published:** 2021-01-26

**Authors:** Tharu Tharakan, Rong Luo, Channa N Jayasena, Suks Minhas

**Affiliations:** 1 Section of Investigative Medicine, Imperial College London, Hammersmith Hospital, London, United Kingdom; 2 Department of Urology, Imperial Healthcare NHS Trust, Charing Cross Hospital, Fulham Palace Road, London, United Kingdom

**Keywords:** Male infertility, azoospermia, genetics, artificial intelligence, sperm retrieval

## Abstract

Infertility affects 1 in 6 couples, and male factor infertility has been implicated as a cause in 50% of cases. Azoospermia is defined as the absence of spermatozoa in the ejaculate and is considered the most extreme form of male factor infertility. Historically, these men were considered sterile but, with the advent of testicular sperm extraction and assisted reproductive technologies, men with azoospermia are able to biologically father their own children. Non-obstructive azoospermia (NOA) occurs when there is an impairment to spermatogenesis. This review describes the contemporary management of NOA and discusses the role of hormone stimulation therapy, surgical and embryological factors, and novel technologies such as proteomics, genomics, and artificial intelligence systems in the diagnosis and treatment of men with NOA. Moreover, we highlight that men with NOA represent a vulnerable population with an increased risk of developing cancer and cardiovascular comorbodities.

## Introduction

Infertility affects 1 in 6 couples, and male factor infertility has been implicated as a cause in 50% of cases^1^. Azoospermia is defined as the absence of spermatozoa in the ejaculate and is considered the most extreme form of male factor infertility. Historically, these men were considered sterile but, with the advent of testicular sperm extraction and assisted reproductive technology (ART), men with azoospermia are able to biologically father their own children. This review provides an up-to-date summary of current techniques used in the management of non-obstructive azoospermia (NOA) and highlights future areas of research.

### Definition and aetiology

Azoospermia is classified as NOA or obstructive azoospermia (OA). NOA occurs when there is an impairment of spermatogenesis, whilst OA is caused by occlusion of the testicular and genital ductular system. NOA has been estimated to affect 1 in 100 men^2^.

**Figure 1.**
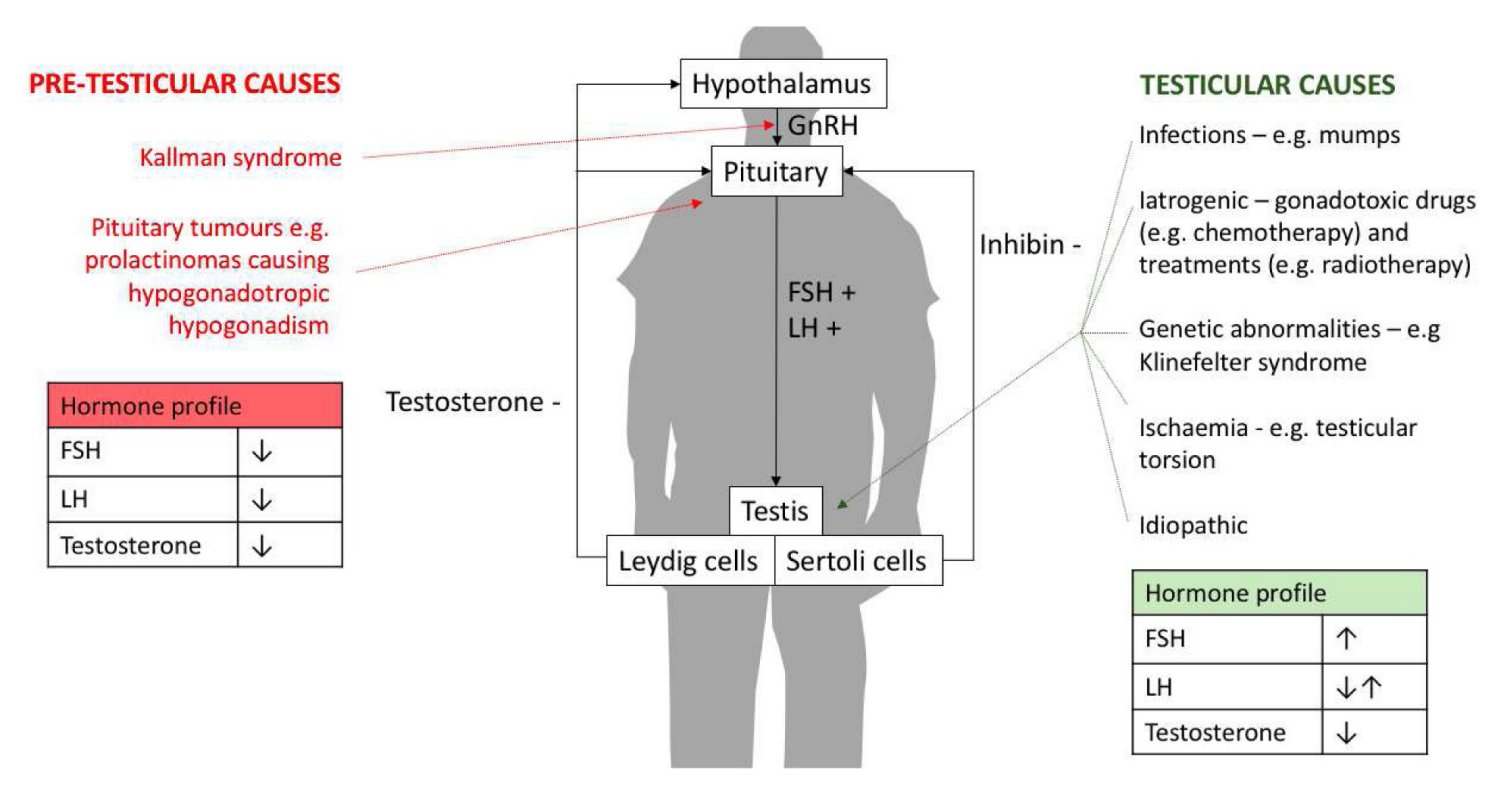
The causes and male reproductive hormone profiles associated with non-obstructive azoospermia. FSH, follicle-stimulating hormone; GnRH, gonadotropin-releasing hormone; LH, luteinising hormone.

The aetiology of NOA is conventionally categorised by the anatomical position of the cause: pre-testicular or testicular. Pre-testicular NOA (or secondary hypogonadism) arises because of a hormone abnormality such that a structurally normal testis is not stimulated effectively to produce sperm usually secondary to hypothalamic-pituitary disorders. Testicular azoospermia (or primary hypogonadism) relates to an intrinsic defect in the testicles leading to impaired spermatogenesis (Figure 1).

The histological features of NOA are classified into hypospermatogenesis, maturation arrest, and Sertoli-cell only (SCO). In hypospermatogenesis, germ cells of all stages of spermatogenesis are present but with a relative paucity in numbers. In maturation arrest, spermatogenesis is incomplete and halts at primary or secondary spermatocyte (early) or spermatid (late) stages. Therefore, mature spermatozoa are usually absent. SCO syndrome is characterised by a complete loss of germinal epithelium. It must be appreciated that in men with NOA, mixed histological patterns are common^3^.

## Management of NOA

### Fertility aspects

The use of gonadotrophin therapy is well established in pretesticular NOA (hypogonadotropic hypogonadism) and has been associated with natural conception within 6–9 months (although up to 24 months has been reported) from the onset of treatment^4^. There have been several retrospective case reports^5–7^ and case series^8,9^ and a limited number of case control studies^10–14^ supporting the use of hormone stimulation in men with primary testicular NOA. Hussein *et al*. performed a prospective study of 612 patients with NOA and treated 496 men in the intervention group with clomiphene prior to sperm retrieval. The authors observed that 10.9% of men who received clomiphene had sperm in the ejaculate and did not require further surgical intervention^10^. However, the current literature is limited owing to heterogeneous methodology in terms of drugs used, treatment protocols, and cohort of patients. Furthermore, contemporary evidence is of low quality, with a paucity of randomised controlled trials. Therefore, the European Association of Urology (EAU) guidelines^15^ do not advocate the use of hormone therapy in men with NOA and primary hypogonadism, and the mainstay of treatment is testicular sperm extraction.

Historically, testicular sperm aspiration (TESA) and extraction (cTESE) involved random biopsies of testicular tissue. However, Schlegel pioneered the use of optical magnification to target specific seminiferous tubules that were more likely to contain spermatozoa^16^. These tubules are classically larger in size and more opaque than the majority of surrounding ones. An advantage of the use of optical magnification is it offers direct visualisation of testicular vessels and therefore minimises the risk of inadvertent vascular damage and potential hypogonadism. Consequently, microdissection testicular sperm extraction (mTESE) has been adopted as the optimal technique of surgical sperm retrieval. Schlegel reported that mTESE, compared with cTESE, removed far less testicular tissue (average mass of tissue removed in mTESE is 1.3% of cTESE) and was associated with a higher rate of successful sperm retrieval (50–60% in mTESE versus 20–45% in cTESE)^16^. Similarly, Amer *et al*. observed that mTESE had a significantly higher sperm retrieval rate than cTESE (47% versus 30%, *P* <0.05) despite a significantly lower volume of testicular tissue excised (4.65 mg versus 53.57 mg, *P* <0.05)^17^. However, it must be noted that within the literature there is a paucity of studies comparing the amount of testicular tissue excised between cTESE and mTESE.

Bernie *et al.* performed a meta-analysis that compared the sperm retrieval rates in mTESE, cTESE, and TESA and observed that mTESE had the highest successful sperm retrieval rate. The authors also noted that mTESE had a 1.5-fold higher sperm retrieval rate than did cTESE and cTESE had a 2-fold higher rate of sperm retrieval than did TESA^18^. Successful sperm retrieval was associated with testicular histology, and hypospermatogenesis was observed to have the highest sperm retrieval rate (73–100%), followed by late maturation arrest (27–86%), early maturation arrest (27–40%), and finally SCO (22–41%)^19,20^. Surgical and biological factors have also been reported to influence sperm retrieval outcomes. Ishikawa *et al*. reported a higher sperm retrieval rate in a surgeon’s second 50 mTESE procedures compared to their first 50 mTESE procedures (44% versus 32%, *P* <0.05)^21^. Furthermore, Modarresi *et al*. observed that the additional use of enzymatic digestion (e.g. collagenase) yielded sperm in approximately 9% of cases where conventional mechanical extraction of sperm from testicular tissue had failed^22^. This suggests that there is a learning curve for both mTESE and also biological techniques, but it must be appreciated that surgical complications such as haematoma, fibrosis, and testicular atrophy have been reported to be less frequent in mTESE compared to cTESE^20^. In this setting, mTESE is considered the gold standard for surgical sperm retrieval, but it must be recognised that this technique does require more specialist expertise, longer procedure times, and higher operating costs compared to cTESE or TESA^20^. Whilst histological subtype and surgical method have been reported to be predictors of successful sperm retrieval, there is insufficient evidence to support both factors’ use to discriminate between NOA men who should undergo surgical sperm retrieval surgery and those who should not^23^.

An alternative method of approaching surgical sperm retrieval in NOA is through identifying areas of spermatogenesis within the testicle via fine-needle aspiration (FNA). This process is known as testicular mapping^24^ and involves FNA at pre-determined sampling sites. The cytological results of testicular mapping are used to generate a geographical “heat map” of where spermatogenesis is present and subsequently used to guide further surgical sperm retrieval procedures in the form of either cTESE or mTESE at a secondary procedure. The advantages of testicular mapping are that it offers a methodical approach of assessing the whole testicle, thereby possibly reducing the likelihood of missing any areas of spermatogenesis. Beliveau and Turek^24^ reported in a cohort of 159 men with NOA who underwent testicular mapping a 90% chance of successful sperm retrieval using directed TESE in men who had 2 or more sites of spermatogenesis on a 18 sites/testicle map^24^. However, testicular mapping has been criticised because it does not extract viable sperm for intracytoplasmic sperm injection and therefore patients will need to undergo two separate procedures when only one may be required. Moreover, the need for two procedures will inevitably result in delays in subsequent ART procedures, which could prove detrimental given that increasing female age is associated with poorer outcomes^25^. The methodical value of mapping through FNA is also questionable, as, arguably, the systematic examination of a testicle under optical magnification with mTESE would achieve similar aims but with the additional yield of viable sperm, although randomised controlled studies are needed to substantiate this. Indeed, there is a paucity of studies investigating the use of FNA mapping in comparison to cTESE or mTESE, and there is also an absence of any cost–benefit analysis. In this setting, FNA mapping cannot be routinely advocated and further large-scale prospective randomised controlled trials are needed.

### Non-fertility aspects

There is increasing evidence that male infertility is not only a risk factor for a decreased life expectancy and malignancy but also associated with a higher rate of cardiovascular comorbidities^26^. With reference to NOA, there are data showing that the prevalence of testicular cancer is 10.5% in men with SCO syndrome^27^. Furthermore, Eisenberg *et al*. observed that azoospermic men (both NOA and OA) had a 2.2-fold increased risk of cancer compared to non-azoospermic men, with men younger than 30 being most susceptible^28^. Whilst the cause for this predisposition to cancer may be representative of the underlying aetiology of NOA (i.e. cryptorchidism confers an increased risk of testicular cancer^29^ and Klinefelter syndrome patients are at increased risk of lung and breast cancer^30^), in many cases the cause remains unknown. In such cases of idiopathic NOA, genetic factors have been postulated to be not only causative to the condition but also contributory to the increased susceptibility to cancer. In animal studies, it has been reported that abnormalities in DNA repair genes result in both azoospermia and malignancy^31^. Indeed, Anderson *et al*. observed in a retrospective cohort study consisting of over 25,000 men that both first- and second-degree relatives of azoospermic men had a significantly increased risk of thyroid cancer compared with fertile men^32^. Whilst not specific to NOA, there are data linking the increasing incidence of testicular cancer, hypospadias, cryptorchidism, and male infertility secondary to *in utero* exposure to environmental endocrine-disrupting chemicals^33^. This testicular dysgenesis syndrome may explain the association between testicular cancer and male infertility but not the association with other cancer subtypes, and further studies are needed to better characterise the association between NOA men and malignancy.

It is also important to recognise that because of the association of NOA with hypogonadism, this cohort of patients are at risk of osteoporosis, type 2 diabetes, obesity, and depression^34^.

Therefore, NOA men represent a vulnerable population with an increased risk of developing cancer and cardiovascular comorbodities. Within this context, clinicians must take a holistic approach when assessing NOA men to exclude other medical issues alongside the treatment of infertility, and it would seem logical to screen for testicular cancer by ultrasound and counsel these men regarding red flag symptoms and signs to prompt early referral. Similiarly, these patients should be screened for symptoms of hypogonadism, as they may be candidates for future testosterone replacement therapy. Indeed, the EAU guidelines recommend that all NOA patients should undego a comprehensive assessment, as this allows the identification and treatment of other comorbidies^35^.

## Emerging technologies and investigations

In spite of the aforementioned advances in surgical techniques, the successful sperm retrieval rate is between 40 and 60% in men with NOA^16^. Within this context, novel technologies have been trialled to optimise sperm retrieval in NOA. There has been increasing interest in the ability to precisely and rapidly identify areas of focal spermatogenesis during testicular sperm extraction, although the majority of these studies are limited to *ex vivo* tissue and *in vivo* rodent studies.

Multiphoton microscopy (MPM) uses low energy infrared femtosecond pulsed lasers to induce excitation of molecules. Intracellular fluorophores such as NADPH, flavins, retinol, and tryptophan and its derivatives absorb energy from laser photons to become excited^36^. As it returns back to its ground state, the energy is released as autofluorescence. Combined with second harmonic generation by supramolecular structures, MPM can produce highly detailed imaging of deep underlying tissues (up to 400 μm) in real time with reduced damage to superficial tissues. In an *ex vivo* human testis study (n = 7), concordance between MPM and traditionally stained slides was 86%^37^. Ramasamy *et al.* used MPM to identify different stages of spermatogenesis in rodent testes via a single median incision similar to that of mTESE, noting differences in fluorescent attributes between tubules with spermatogenesis and those without^38^. Therefore, MPM could potentially optimise surgical techniques to identify tubules with spermatogenesis intraoperatively prior to making any biopsies and reduce the total number of testicular excisions necessary and subsequent risks of testicular atrophy and hypogonadism. However, there are significant barriers in current MPM technology before it can be applied to clinical use. MPM has a narrow field of view^39^, approximately 200 μm in diameter, which is about the diameter of a single seminiferous tubule. At 4 frames per second, processing each tubule individually is tedious and would be subject to motion artefacts during *in vivo* applications, especially using portable MPM prototypes designed for intraoperative use^40^, although some mitigation is possible through an inbuilt tracking mechanism to compensate for the patient’s movements^41^. Moreover, there are concerns over the mutagenic potential of lasers on spermatozoa^37^. A low power setting would be safer for spermatozoa but renders dim images that cannot be accurately interpreted^37^. In addition to this, the use of lasers in MPM would warrant extra safety precautions such as specialist theatres and laser safety goggles that offer protection against retinal damage^42^. In addition, the invisible laser can be reflected unwittingly through reflective surfaces such as intraoperative surgical tools, rings, or glassware^42^.

Full field optical coherence tomography (FFOCT) uses white light interferometry to precisely examine the tomographic surface of a tissue specimen. The specimen is illuminated by a simple white light source, and optical beams of multiple wavelengths are reflected differentially depending on the subsurface features of the sample. The diffraction patterns, also known as waves, are used to decipher histology in real time (within a few minutes) without traditional fixing, staining, and processing, which is time consuming and tissue destructive. Currently, OCT technology is widely used in ophthalmology for the visualisation of the retina, but its ability for precise, non-invasive diagnostics lends itself to wider applications. In a pilot rodent study, FFOCT was successfully used to identify spermatogenesis in freshly excised testicular tissue^43^. The sensitivity and specificity of FFOCT are 80% and 95%, respectively, of equivalent histological diagnosis^44^.

However, unlike MPM, FFOCT is limited to “en-face” superficial imaging with limited depth. Beyond the depth of 1–2 mm, light becomes scattered instead of being reflected. There is also an absence of cellular details to give structural context to the light signals produced by sperm tails^43^. However, the use of white light at eye-safe levels makes this process highly unlikely to cause damage to spermatozoa, especially since halogen lamps are already in use in operating rooms^43^. In a mTESE procedure, the freshly excised testicular tissue can be additionally scanned via FFOCT to confirm the presence of spermatogenesis intraoperatively prior to closing up the main incision. Almog *et al.* developed an endoscopic FFOCT system to perform *in vivo* neuroimaging experiments of a rat brain^45^, although no similar studies have yet been reported on testicular tissue.

Raman spectroscopy (RS) utilises the interaction of light with chemical bonds within a tissue sample to generate a spectra graph for each molecular structure. The unique scattering of light allows for distinction between different tissue types and identification of tubules with spermatogenesis. This has been demonstrated in *ex vivo* rodent and human studies, with a reported high specificity and sensitivity^46,47^. However, in contrast to the direct visual imaging techniques discussed above, RS relies on analytical interpretation of spectra results to deduce if spermatogenesis is present and to what extent. This therefore requires the creation of a customised algorithm that is able to reliably interpret different patterns of spectra peaks and correlate with underlying histology. Given the complexity of mixed histological patterns seen in NOA and the wide variation of cellular makeup of each individual, the creation, testing, and refining of such an algorithm is challenging.

## Novel genetic studies

There is an impetus to understand the genes that contribute to the pathogenesis of NOA in order to help our understanding of the disease process and also to identify potential predictors for successful sperm retrieval. Spermatogenesis is a highly sensitive process and can be adversely affected by both environmental and genetic factors. The utilisation of mouse models has allowed the identification of more than 473 genes that contribute to male infertility^48^.

Currently, the use of chromosomal karyotype assays for Klinefelter syndrome and polymerase chain reaction analysis for azoospermia factor (AZF) microdeletions are established in clinical practice. Both of these tests are useful for diagnosing the underlying cause of NOA and critical for fertility counselling given that all Y chromosomal mutations will be inherited in future male offspring.** With the exception of the AZF microdeletion, which can be found in 3–10% of men with NOA varying with population geography^49,50^, the frequency of the remaining monogenic mutations are too low for routine clinical testing. Men with complete AZFa or AZFb microdeletions have extremely poor sperm retrieval outcomes^51,52^. The sperm retrieval rate for AZFc microdeletions range between 33 and 87% owing to their wide phenotypic presentations^53,54^ (Table 1).

**Table 1.** Genes or proteins that have been correlated with sperm retrieval rate.

Name	Details	SRR outcomes	Additional information	Source
Genetic markers using RT-PCR on testicular biopsies				
AZF	AZFa – *USP9Y* and *DBY*	Very poor	Complete AZFa deletion causes Sertoli-cell only syndrome (SCOS) Deletion of USP9Y only results in hypospermatogenesis	51 54
	AZFb – *RBMY* and *PRY*	Very poor	Corresponds with maturation arrest in histology	^52^
	AZFc – 12 genes	33–87%	Common, wide phenotypic ranges from SCOS to normospermatogenesis	53,54,56
JMJD1A	Jumonji domain-containing 1a	Positive	n = 22 Sensitivity 91%, specificity 89%	57
RNF8	E3 ubiquitin ligase Chr 6p21.3	Positive	n = 47 Sensitivity 81%, specificity 84%	58
SPEM1	Post-meiotic marker, testes- specific gene	Positive	n = 63 Sensitivity 96%, specificity 85%	59
VASA	Chr 5q, expressed specifically in germ cells	Positive	n = 52 Sensitivity 87%, specificity 86% Independent predictor after multivariant analysis	60
Protein markers in seminal plasma				
Clusterin	Apolipoprotein J, secreted by Sertoli cells	Positive	Seminal Clusterin in conjunction with FSH levels associated with successful TESE (n = 89), with reduced risk of abnormal spermatozoa and DNA fragmentation, although this may be confounded by hypertension	61 62 63
LGAL3BP	Lectin galactoside-binding, soluble 3 binding protein	Positive	Seminal LGAL3BP is higher in men with successful TESE n = 40 Sensitivity 100%, specificity 45%	64
HE1	Epididymal secretory protein E1, cholesterol transporter	Positive* IVF	Higher levels associated with successful IVF n = 13	65
TEX101	Cell membrane protein exclusive to testicular germ cell	Positive	n = 137 Sensitivity 73%, specificity 64% Proposed use of two marker algorithm ECM1 and TEX101	66 67

FSH, follicle-stimulating hormone; IVF, *in vitro* fertilisation; RT-PCR, real-time polymerase chain reaction; SRR, sperm retrieval rate; TESE, testicular sperm extraction.

Beyond the identification of causal genes in NOA, comparative genomic and proteomic studies have been utilised to discover novel predictors of successful sperm retrieval. The majority of studies compare differential expression of selected genes or proteins in testicular biopsy samples or seminal plasma (Table 1). However, seminal plasma has been adopted as the preferential choice because it can be obtained non-invasively and may more accurately reflect the microenvironment of the testes.

Whilst the above genetic markers have been reported to discriminate between successful and unsuccessful sperm retrieval, this does not necessarily equate to successful clinical pregnancy. Indeed, Dorosh *et al.* investigated the expression of markers associated with the final stages of spermatogenesis, ACR and GAPDHS, and observed that, despite high fertilisation rates of NOA patients with these markers (71% and 66%, respectively), clinical pregnancy was lower (6% and 8%, respectively) than the reported average NOA cohort (26%)^68^.

Currently, the single most reliable predictor of sperm retrieval in NOA is histopathology. The presence of hypospermatogenesis was associated with higher rates of sperm retrieval (94%) than maturation arrest (37%)^19,69^. The lowest sperm retrieval rates are associated with a histological pattern of SCO syndrome (24%)^70–72^. In this regard, the predominant histological subtype may be used to counsel men regarding the likelihood of a successful secondary surgical sperm retrieval procedure, although this has no value as a prognostic tool in predicting testicular sperm extraction at the time of a primary procedure.

Other non-invasive markers such as clinical and hormone parameters have been investigated as potential predictors of successful sperm retrieval (Table 2). However, the evidence for biochemical and clinical markers is conflicting. Serum hormone levels have been reported to be inaccurate markers of surgical sperm retrieval rates because they represent global testicular function rather than reflect the heterogenicity of testicular tissue^92^.

**Table 2.** Summary of biochemical and clinical predictors of sperm retrieval in non-obstructive azoospermia.

Hormonal factors	
Follicle- stimulating hormone (FSH)	FSH is secreted by the pituitary to stimulate Sertoli cells, which support the spermatogenic process of germ cells in men. An elevated FSH level has been associated with impaired spermatogenesis (Figure 1). Hence, some studies have proposed that a high level of FSH could be a predictor of SRR in mTESE^73,74^. Yang *et al.* performed a meta-analysis of 11 studies that investigated FSH as a predictor of SRR prior to cTESE/mTESE. The AUC value of 0.72 was obtained after pooled analysis, suggesting FSH to be a moderate predictor of SRR^75^. In contrast, Corona *et al*.^76^ reported no correlation between FSH levels and SRR after pooling 117 studies. These inconsistencies in the literature may be explained by differences in surgical technique given that Yang’s meta-analysis included studies predominantly using cTESE (20/21), whilst Corona’s meta-analysis included 56 studies using cTESE and 43 studies using mTESE. Indeed, Ramasamy *et al*. observed in a large retrospective study of 792 men with NOA undergoing mTESE that SRRs were not associated with FSH levels^77^.
Inhibin B	Inhibin B is produced by Sertoli cells and acts as a negative feedback regulator of FSH secretion. Therefore, inhibin B is a marker of spermatogenesis. High inhibin B levels in the serum or seminal plasma have been proposed to be an independent predictor of SRR^78^. In a cohort of 403 NOA men, higher inhibin B levels were associated with successful SRR. The reported sensitivity was 77.9% and specificity was 91.58% (mixed cohort of mTESE and cTESE)^79^. Yet Vernaeve *et al.* observed in a cohort of 185 NOA men that inhibin B was a poor discriminator of successful SRR (cTESE) with a sensitivity of 44.6% and a specificity of 63.4%. Using ROC analysis, the authors observed an AUC of 0.51 for the inhibin B concentration of 13.7 pg/ml^80^. Moreover, a meta-analysis comprising 32 studies reported that serum inhibin B had a sensitivity of 0.65 and a specificity of 0.83 for predicting sperm retrieval in cTESE^81^.
Composite markers	Given the conflicting data regarding the utility of FSH and inhibin B individually as predictors of SRR, there have been attempts to combine both hormone levels to increase the predictive power of these markers. Von Eckardstein *et al.*^82^ observed that together serum FSH and inhibin B levels were a more sensitive predictor of the state of spermatogenesis than alone. However, collectively, these hormones predicted SRR from cTESE with a sensitivity of 75% and specificity of 73%. Similarly, Vernaeve *et al.* performed a ROC analysis utilising both inhibin B and FSH and reported that the AUC of inhibin B in men with an FSH level of <25 and ≥25 IU/l (the best threshold value for discriminating successful and unsuccessful cTESE was 25 IU/l) was 0.53 and 0.50, respectively^80^. Boitrelle *et al.* combined testicular volume with serum FSH and inhibin B levels in a tri-variable predictive algorithm to predict cTESE outcomes^83^. The authors reported that a score utilising these parameters was able to predict a successful cTESE with a positive likelihood ratio of +3.01. A score value of less than 18.5 correlated with a successful cTESE in 77.4% of cases and live birth rate in 41.8% of cases. Moreover, this value was also predictive of a sperm yield of greater than 100 spermatozoa in 91.1% of cases.
Summary	Within the context of the current literature, we cannot advocate a specific biochemical marker as a predictor of successful SRR owing to conflicting data.
Clinical factors	
Patient age	Amer *et al*.^17^ investigated predictors of “difficult” (those with long durations, multiple biopsies required, and reduced SRR) sperm retrieval operations (mixture of cTESE and mTESE). The authors reported that histological pattern, FSH level, and testicular volume were not attributable. However, older age was a significant predictor and the mean age (SD) in more difficult operations was 39.4+/–7.95 compared to 32.75+/–6.76 (*P* <0.05) in those deemed less difficult. Moreover, the duration of infertility was noted to be a significant discriminator with the more difficult operations having a mean (SD) duration of 9.8+/–6.1 years compared to 5.2+/–3.8 years in those less difficult (*P* <0.05). Gnessi *et al.* observed that younger age was predictive of shorter procedure duration and faster recovery time. However, this study reported that age was not predictive of cTESE outcome after multivariable analysis^84^.
Smoking	In a prospective study of 64 NOA men undergoing cTESE, tobacco use was observed to be an independent negative predictor of SRR (odds ratio 0.269, *P* = 0.045)^85^.
Testicular volume	Testicular volume has been reported to be a predictor of SRR. Corona *et al*. observed in a meta-analysis comprising 117 studies (both mTESE and cTESE) that testicular volume was the only significant predictor of successful SRR. ROC curve analysis for a testicular volume of >12.5 ml predicted a SRR of >60%^76^. However, caution must be applied to the above finding, as severe testicular atrophy does not exclude successful mTESE outcomes. Bryson *et al.* reported a SRR of 55% with a testicular volume of <2 ml, 56% with a testicular volume of 2–10 ml, and 55% with a testicular volume of >10 ml in 1,127 patients^86^.
Cryptorchidism	A history of cryptorchidism was not associated with SRR in NOA men. Raman and Schlegel reported that the SRR (mixed cohort of mTESE and cTESE) was 74% in the cryptorchid cohort (n = 35) and 58% in the non-cryptorchid cohort (n = 274)^87^. Barbotin *et al*. observed no significant difference in SRR for cTESE between unilateral (60%) or bilateral cryptorchidism (66.2%), *P* = 0.353^88^.
Procedural factors	
Previous failed TESE attempts	Friedler *et al*. reported that, in repeated cTESE procedures, the successful SRR was 33/39 (85%) and sperm could be found in men undergoing their fifth cTESE. Furthermore, there were no differences in fertilisation, implantation, or clinical pregnancy rate from sperm acquired from the first procedure and subsequent procedures^89^. Moreover, Kalsi *et al*. observed that repeat mTESE had no impact on SRR, as 40% of those with previous failed cTESE or TESA procedures were successful on repeat attempts^90^.
Embryological factors	The embryological extraction process has been reported to impact on SRR. Studies have reported that the addition of enzymatic treatment coupled with the conventional mincing method of testicular tissue confers a higher sperm yield^22,91^.
Surgical factors	There is evidence that there is a learning curve for mTESE. Ishikawa *et al*. compared the SRR for a surgeon’s first 50 mTESE procedures compared to the subsequent 50 procedures. There were no differences in the patient clinical factors between the two cohorts, but the operation times were shorter and the SRR was higher in the later operation group (*P* <0.05)^21^.

AUC, area under the curve; cTESE, conventional (open) biopsies testicular sperm extraction; mTESE, microdissection testicular sperm extraction; NOA, non-obstructive azoospermia; ROC, receiver operating characteristic; SD, standard deviation; SRR, sperm retrieval rate; TESA, testicular sperm aspiration.

## Artificial intelligence in male fertility medicine

The emergence of increasingly sophisticated computer technology combined with larger digital storage has allowed the generation of novel data acquisition. This large quantity of data requires processing in a timely and meaningful manner. Artificial intelligence (AI) is a branch of computer science that has been incorporated to assist in data analysis and can discern patterns among complex variables and formulate predictions through algorithms.

Recently, AI has been successfully applied in various medical fields that rely on image-based investigations such as radiology, gastroenterology, and oncology. In radiomics, AI was able to index patterns within medical imaging and apply algorithmic rules to accurately classify different radiographic patterns of disease^93^. This includes not only the diagnosis of pathology but also prognosis and therapeutics^94^. Miotto, Kidd, and Dudley were able to use AI to predict the development of future medical conditions such as severe diabetes, schizophrenia, and various cancers in their patient population based on electronic heath records^95^. Gil *et al*. successfully used AI decision trees to predict semen quality in men based on environmental and lifestyle factors obtained through simple questionnaires with a reported accuracy of 86%^96^. Such tools could be useful for identifying men who are at risk of infertility and allow early targeted interventions such as lifestyle advice in a primary care setting. Many men with infertility were unaware of the impact of their adverse lifestyle decisions on their fertility^97^.

The role of AI in male infertility can be broadly classified into two areas: predictive modelling to enhance clinical decision making and optimisation of surgical procedures.

## Predictive modelling

Predictive modelling may allow stratification of men who are more likely to have successful sperm retrieval prior to an mTESE procedure. Despite advances in sperm retrieval techniques, the success rate is only 50%^76^, and we are unable to reliably predict, and therefore counsel, which patient will likely benefit from surgery. Whilst there are inconsistencies in the literature regarding a single biomarker, it may be that a composite of two or more markers may be a more accurate predictor. Several studies have used multivariable regression analysis to develop predictive modelling to discriminate surgical sperm retrieval success. Cissen *et al*. reported that a prediction model based on several variables (male age, serum levels of luteinising hormone [LH], follicle-stimulating hormone [FSH], and testosterone, the diagnosis of idiopathic NOA, and the presence of an AZFc gene deletion) had an area under the receiver-operating characteristic (ROC) curve (AUC) of 0.69. Moreover, the model was validated and identified to have a moderate discriminative value (AUC: 0.65). However, the authors noted that the study cohort had a disproportionately lower number of Klinefelter syndrome patients owing to governmental restrictions and thus there was selection bias^98^. Ma *et al*.^99^ developed a multivariable logistic model to predict the likelihood of sperm retrieval failure using TESA in NOA men. The authors reported that the predictive model had an AUC of 82.3% with a sensitivity of 0.677 and a specificity of 0.863. However, this study was limited because some of the FNA results were misclassified.

AI has been utilised to rapidly analyse data and identify complex patterns. This has been tested by Ramasamy *et al.* using artificial neural networks (a new multivariate statistical model) to formulate new algorithms to predict sperm retrieval outcomes using a training set of 770 patients^100^. The information included was confined to clinical and biochemical markers such as FSH, age, and testicular volume, and the authors reported an accuracy of 59%^100^.

Chen and colleagues used artificial neural networks to predict sperm retrieval rates utilising 12 variables (testicular volume, semen volume, seminal pH, seminal alpha glucosidase and fructose, serum FSH, LH, total testosterone, prolactin, oestradiol, and serum and seminal leptin^101^). The patient cohort comprised 280 NOA men undergoing cTESE. Three artificial neural network models were constructed and were observed to be a more accurate discriminator of successful sperm retrieval rate than FSH or leptin alone. Moreover, using ROC curve analysis, one of these models had an AUC of 0.83. However, whether the dataset size is large enough to allow accurate estimation of 12 variables is uncertain.

Zeadna *et al*. investigated the predictive capacity of models developed by machine learning (gradient boosted decision trees) and standard multivariate logistic regression to determine cTESE success. The variables studied were FSH, LH, testosterone, semen volume, age, body mass index, ethnicity, and testicular size^102^, and ROC curve analysis showed an AUC for the gradient boosted trees of 0.81, whilst the AUC for the multivariate logistic regression model was 0.75. However, the study was limited because it included a dataset consisting of only 119 patients.

More robust data sets, including the latest relevant genetic, proteomics, lifestyle, and environmental data, may allow AI algorithms to predict NOA men who are likely to develop malignancy and therefore help stratify a potential NOA screening population. This would result in obvious benefits in patient counselling and more effective use of health resources.

## Procedural optimisation

AI and automation can simplify and expedite procedures. It can be a cost-effective way to boost standardisation of a process by eliminating human bias and reducing workforce. In the diagnostic workup for male infertility, semen analysis is performed according to WHO reference guidelines. In current practice, computer-aided sperm analysis (CASA) systems are able to report on motile percentage and kinematic parameters, allowing for high-throughput analysis^103^. Thirumalaraju *et al*. developed an artificial neural network that was reported to be able to accurately discriminate normal and abnormal sperm morphology with a sensitivity and specificity of 100%^104^. However, this was tested in a sample size of only nine^104^.

Robotic surgery represents another AI application in clinical medicine. The da Vinci robotic system allows for precision in microsurgical procedures, given that the instrumental arms offer an ability to rotate 540^o^, beyond the natural limits of a human hand. The robotic system also allows for high-resolution magnification. Although more commonly applied in laparoscopic abdominal surgeries, the technology can also be adapted in NOA. Parekattil and Gudeloglu performed 12 robotic-assisted mTESE without any complications^105^. A robotic platform would also be more amenable to incorporate modern imaging modalities such as MPM or FFOCT intraoperatively, overcoming some of the logistical barriers. Moreover, a robotic system could also offer virtual training modules^106^, telementoring, and long-distance telesurgery^107^. However, robotic systems come at a high expense in terms of both initial purchase and maintenance costs^107^.

## Conclusion

The advent of testicular sperm extraction techniques has allowed men with NOA to father biological children. Whilst our understanding of the genetic and environmental mechanisms that underpin this disease expands, further research is needed to understand how to optimise surgical sperm retrieval rates and also discriminate between those in whom spermatogenesis is present and those in whom spermatogenesis is absent. Furthermore, it is now recognised that men with NOA are a vulnerable population that are at a higher risk of cancer and also other medical comorbidities. Therefore, the management of NOA should be holistic and address conditions outside the remit of male infertility.

Currently, there are significant inconsistencies in the literature regarding the role of both clinical and biochemical predictors of sperm retrieval outcomes, and further prospective, multi-centred trials are urgently required. There are emerging data highlighting the potential benefits of AI technologies in processing large collections of data to formulate algorithms that can be used to guide clinical management. Therefore, AI represents a promising tool to predict outcomes in NOA men and help patient counselling and healthcare resource management.
